# Challenges in the management of extremity vascular injuries: A wartime experience from a tertiary centre in Sri Lanka

**DOI:** 10.1186/1749-7922-6-24

**Published:** 2011-08-10

**Authors:** WDD de Silva, RA Ubayasiri, CW Weerasinghe, SM Wijeyaratne

**Affiliations:** 1 University Surgical Unit, National Hospital of Sri Lanka, No. 1, Regent Street, Colombo 10, Sri Lanka

## Abstract

**Background:**

Management of peripheral vascular injuries often present critical challenges in resource limited settings of developing countries. The additional burden from a military conflict poses further challenges. Delays in presentation often result in the loss of limb and even life, in what is usually a young active population. The objective of this report is to analyse the early outcome of vascular intervention at a tertiary referral centre in Sri Lanka.

**Methods:**

A retrospective descriptive review of eighty one consecutive extremity vascular injuries in seventy patients during a seven month period was performed with regards to the cause of injury, types of presentations, ischaemia time, interventional procedures, complications and early outcome.

**Results:**

Mean age was 31.2 years (9-72 years) and 96% were males. Injuries were caused by blasts in 41%, cuts in 26%, gunshots in 17% and road traffic injuries in 9%. Indications for revascularization were acute ischaemia in 44%, active bleeding in 43% and pseudo-aneurysms in 13%. Six patients underwent primary amputations due to non-viable limbs. 64 patients underwent vascular intervention. Fifty one percent needed vein grafts while 46% had direct repairs. Bleeding was often (73%) from upper extremity injuries. Median time to revascularization was 5.5 (2-16) hours with all limbs salvaged. Acute ischaemia (40%) was often from popliteal injuries. Median time to revascularization was 10 (5-18) hours and viability was prejudged at fasciotomy. 92% of revascularized limbs were salvaged. There was no perioperative mortality.

**Conclusions:**

Results from vascular repairs are encouraging despite significant delays.

## Background

Vascular injuries accounts for 2-3% of civilian trauma [1-3] and around 7% of combat related trauma [4]. Early intervention is considered crucial for successful outcomes. The recent military conflict in Sri Lanka saw an exponential rise in the number of vascular injuries. The extra volume and injury complexity due to the military conflict was an add-on to the pre-existing civilian trauma service. Limited facilities to manage vascular injuries in most parts of Sri Lanka coupled with delays in diagnosis and transfer to tertiary care centres, pose major challenges with regards to optimum management of these injuries. Such limitations would be seen in most parts of the world, even those without military conflicts and lessons learnt in Sri Lanka may be applicable in general. We report on the causes of injury, type of presentation, repair methods, treatment delay and early outcome in relation to vascular injuries presenting to the University Vascular Unit in Colombo, Sri Lanka.

### Patients and Methods

Seventy consecutive patients presenting to the University Vascular Unit in Colombo with extremity vascular injuries during a seven month period were studied. Interventions included both surgical and endovascular techniques. Data was prospectively entered in to a database for retrospective analysis. Time to revascularization was defined as the period from the approximate time of injury to the time at which the patency of the injured vessel was restored at surgery. Limb salvage was defined as the presence of a viable limb at one month after injury, regardless of functional outcome.

Patients either presented directly to the University Surgical Unit via Accident Service, National Hospital or were transferred from peripheral surgical units around the country. All patients were resuscitated according to Advanced Trauma Life Support protocols and then assessed for vascular injury. Assessment was by clinical examination aided by the use of hand held Doppler. In the absence of facilities for emergency contrast angiography at the National Hospital at the time, decisions on surgical exploration and repair were entirely clinical, based on distal ischaemia, pulsatile bleeding, expanding haematoma, palpable thrill or bruit. However in patients presenting with no immediate threat to life or limb such as those with suspected pseudoaneurysms, arterial duplex scanning or angiography was performed before intervention.

When ever a vascular injury presented with limb threatening ischaemia, the decision to proceed with vascular repair as opposed to primary amputation was based on distal muscle viability. This was either clinically evident viz. intact toe or ankle movements, or in instances where such movements were absent or other injuries precluded such testing, open fasciotomy and observation of the contractile response of muscle to direct stimulation was used. Limbs with non-contractile muscle in up to two compartments were considered for revascularization while those with more non contractile muscle were recommended primary amputation in view of the high risk for reperfusion injury and poor functional outcome thereafter. The other considerations prior to vascular repair were the mangled extremity severity score (MESS) score [5] and severity of associated nerve and bone injuries.

Operative exploration of injured vessels was performed via standard incisions and distal and proximal control was obtained. Inflow and backflow were assessed and we routinely passed an embolectomy catheter to proximal and distal segments to perform thrombectomy followed by the flushing of the distal segment with heparinised saline. This was followed by definitive repair. Direct end to end anastomosis was performed if approximation of debrided arterial ends were free of tension. When this was not possible, interposition vein grafting, using autologous reversed long saphenous vein from the contra- lateral limb, was done. A synthetic graft was used only once for an extra anatomical bypass in the case of an external iliac artery injury.

Where venous injury was present, attempt at repair was only made in the case of the axillary, femoral and popliteal veins using either direct repair or vein graft techniques. Other venous injuries were ligated.

Where there were associated bone injuries, orthopaedic fixation followed vascular repair in order to minimize ischaemia time. Nerve injuries identified at the time of surgery were repaired primarily.

Postoperatively the patients were maintained on intravenous prophylactic antibiotics and venous thromboprophylaxis with low molecular weight heparins in the case of lower limb injuries.

## Results

### Demographics

Seventy patients with 81 vascular injuries are evaluated in this report of whom 67 (96%) were males. The mean age was 31.2 years (Range 9- 72) with 75% being less than 40.

### Causes

are tabulated in Table 1. The majority of injuries (41%) were due to high energy blasts from artillery shells and mortars, rocket propelled grenades, high explosive bombs and anti personnel mines. Cuts and stabs accounted for 26% while 17% were due to gunshot wounds. These included high velocity rifles & machine guns; low velocity shot guns and improvised trap guns. Other causes included road traffic accidents (RTA), industrial accidents and iatrogenic trauma following arterial catheterisation. Civilian trauma accounted for 54% of injuries while 46% were related to the military conflict.

**Table 1:** Vessels injured by cause of injury

	*Blast injuries*	*Cuts/stabs*	*Gunshots*	*RTAs*	*Industrial accidents*	*Iatrogenic*	Total (%)
Axillary artery	01		01				02 (2.5%)
Brachial artery	11	01	01	01	03	01	18 (22%)
Radial artery		12					12 (15%)
Ulnar artery		07					07 (8.5%)
Femoral artery	06	01	02	01		02	12 (15%)
Popliteal artery	08		05	04			17 (21%)
Tibial arteries	02		03				05 (06%)
Femoral vein	01		01				02 (2.5%)
Popliteal vein	03			01			04 (05%)
Axillary vein	01		01				02 (2.5%)

	33(41%)	21(26%)	14(17%)	07(9%)	03(3.5%)	03(3.5%)	81 (100%

### Vessels injured and type of presentation

All named extremity vessels presented with injuries and were repaired (table 1). The brachial artery was the most commonly injured vessel (22%) followed by popliteal (21%), femoral (15%) and radial (15%) arteries. Indications for referral were acute ischaemia in 36(44%), bleeding in 35 (43%) and traumatic pseudo-aneurysms in 10(13%). In patients presenting with bleeding, the commonest vessels injured were the radial and ulna arteries (Table 2).

**Table 2:** Presentations and method of management

*Vessel injured*	*Direct repair*	*Vein graft*	*PTFE graft* *bypass*	*Endo-vascular* *stenting*	*Primary* *amputation*	N%
**1. Injuries presenting with bleeding**						
Radial/Ulnar arteries	19					19 (54%)
Brachial artery	01	04				05 (14%)
Femoral artery	01	01				02 (06%)
Axillary artery		02				02 (06%)
Major limb veins	04	03				07 (20%)
***Total***						***35(100%)***

**2. Injuries presenting with acute ischaemia**						
Popliteal artery	03	09			05	17 (47%)
Brachial artery	02	07			01	10(28%)
Femoral artery	01	02	01			04(11%)
Crural arteries		05				05(14%)
***Total***						***36(100%)***

**3. Injuries presenting as psuedoaneurysms**						
Femoral artery	02	03		01		06 (60%)
Brachial artery	01	02				03 (30%)
Popliteal artery		01				01 (10%)
***Total***						***10(100%)***

**Total**	**35**	**39**	**01**	**01**	**06**	**81**

***N.B ***Some patients had multiple repairs.

### Delays in intervention, methods of repair and limb salvage

For injuries presenting with bleeding, median time to revascularization was 5.5 hours (range 2-16) and all limbs were salvaged. In injuries presenting with acute ischaemia, popliteal injuries were the most common (Table 2) and 80% of such limbs were revascularized more than 6 hours after injury. Median time to revascularization was 10 hours with a limb salvage rate was 92% among limbs undergoing revascularization. Fasciotomy was performed in all lower extremity injuries and in 5 out of 9 upper extremity injuries.

Thirty five direct repairs and 39 interposition vein grafts were the most common methods of repair. One synthetic graft bypass and one endovascular stenting for a femoral pseudoaneurysm was also performed (Table 2).

### Primary Amputations

Six patients presenting with ischaemic vascular injuries (5 popliteal, 1 brachial) were found to have non-viable limbs and were offered primary amputation. The delay in presentation ranged from 8 to 20 hours.

### Additional injuries

Eleven patients had concomitant bone injuries and 15 had nerve injuries that were attended to at the same time. Vascular repairs followed open fracture fixation with external devices in 88%. In the remainder where time consuming internal fixation was deemed necessary vascular repairs preceded orthopaedic fixation.

### Complications

There were two secondary amputations, one due to diabetes related sepsis and the other due to graft failure. Infections, deep vein thrombosis, secondary haemorrhage, graft thrombosis were also noted in this series. However there were no cases of clinically detected systemic reperfusion injury and no peri-operative mortality (Table 3).

**Table 3:** Complications

*Complication*	*n*	%
Secondary amputations	02	4%
Wound infection	06	9%
Secondary haemorrhage	01	1.5%
Deep vein thrombosis	03	4.5%
Graft thrombosis	04	6%
Reperfusion injury	00	-
Mortality	00	-

**Total**	16	

## Discussion

The majority of those presenting with vascular injuries are active young men and thus optimal management to control bleeding and re-establish circulation is crucial. The military conflict at the time nearly doubled the vascular trauma workload at our centre which is 6-8 hours away by road from the war zone. The limb salvage rate and overall survival after vascular repair is impressive in this series and compares well with other recent reports. Peck et al reported a secondary amputation rate of 3% and mortality of 1.5% in vascular repairs during operation Iraqi freedom [6]. Velinovic et al described amputation rates of 20% in vascular injuries during the height of the Balkan conflict [7]. In another series, Zohn et al alluded to limb salvage rates of 80% with an all cause mortality of 6% [8].

Our approach to diagnosis by clinical examination alone rather than routine contrast imaging appears effective. Diagnostic arteriography was not available and would probably have caused further delay without adding much to the eventual management decision. Indeed a number of trials have established the primacy of clinical examination over diagnostic arteriography in the diagnosis of vascular injury from both penetrating and blunt trauma in acute situations [9,10]. However we do agree with the recommendation by Ramanathan et al. that arteriography is useful to determine the site of vessel injury in situations where there are multiple external injuries [11].

Although the need for fasciotomy is clear when distal muscles are swollen and tender [2], this is not so when it comes to prophylaxis [12,13]. In this series all patients needing emergency repairs for ischaemia had a fasciotomy to assess limb viability because of delayed presentation and difficulties in assessing neuromuscular function in an injured limb. Compartment pressure measurement may have prevented preliminary fasciotomy in some, but serial measurements would then be necessary to prevent delays in the management of reperfusion induced compartment hypertension. The low threshold for early open fasciotomy in our practice may have contributed to the good outcomes.

The timing of orthopaedic fixation in concomitant bone injury is another source of debate. Prior skeletal fixation is strongly advocated in some series [14,15] while more recent reports have highlighted the importance of reducing ischaemia time by proceeding with vascular reconstruction first [16,17]. Wolf et al reduced ischaemia time by employing temporary shunts and then performing orthopaedic fixation before vascular reconstruction [18]. In our practice, most orthopaedic fixations being external, delays were minimal facilitating vascular repairs on a stable base. In other instances where time consuming internal fixation were deemed necessary the order was reversed.

In our series we observed three patterns of presentation viz. acute ischaemia, bleeding and traumatic pseudoaneurysms. This often had significant implications both on the nature and subsequent course of management. In bleeding injuries the vessels involved mainly those of upper limb vessels and over 60% underwent revascularization before 6 hours. However injuries causing acute ischaemia often presented the real challenge, the majority involving popliteal or femoral vessels with prolonged periods of ischaemia. These were often transferred from peripheral hospitals including those in the war zones. The presence of multiple fragmentation injuries from explosive devices made identification of the site of damage, difficult. Nonetheless, we had a limb salvage rate of 92%. Our policy to revascularize all viable limbs with continued ischaemia in otherwise stable patients even with long periods of ischaemia seems justified. The risk of reperfusion injury has been cited as a reason for conservative management in prolonged ischaemia. However we did not encounter clinically significant systemic effects from reperfusion in this series despite accepting those with non contractile muscles in up to two compartments (Table 3). Similarly, Menakuru describing a series of 148 patients in North India reports excellent results despite a median delay of 9.3 hours in presentation to casualty [19]. This raises an issue regarding the value of "ischaemia time" in predicting outcome and determining intervention. Wagner et al. found a lack of correlation between ischaemia time and outcome in vascular injury [20]. Other authors have pointed out that the severity of tissue ischaemia depends not only on its duration but also on the level of arterial injury, the extent of soft tissue damage and the efficiency of collateral circulation [16]. Additionally, the time since injury, may not necessarily reflect the actual period of ischaemia especially in closed vessel injuries.

This is not to decry that delay in revascularization should not be minimised. Conventional logic dictates that longer the period of ischaemia the higher the chance of limb loss. However to condemn limbs as unsalvageable purely on the basis of ischaemia time alone needs to be reconsidered.

Finally it must be stressed that limb salvage alone is not sufficient and long term functionality which is often dependent upon the extent and recovery from associated neuromuscular and skeletal injuries must be considered in the overall outcome assessment. Nevertheless in Asian societies like ours where physical integrity of limbs often takes precedence over functionality these aspects tend to be overlooked.

## Conclusion

In conclusion, delays in presentation of extremity vascular injuries should not dissuade one from adopting an aggressive approach to repair and limb salvage after pre-procedure fasciotomy to establish muscle viability and pre-empt reperfusion induced compartment hypertension.

## Competing interests

The authors declare that they have no competing interests.

## Authors' contributions

RAU CW & WDD performed the listed procedures, collected the data, performed a literature review and drafted the manuscript. SMW analysed the data and critically revised the manuscript. All authors read and approved the final manuscript.
